# Multidrug-Resistant Pathogens in Burn Wound, Prevention, Diagnosis, and Therapeutic Approaches (Conventional Antimicrobials and Nanoparticles)

**DOI:** 10.1155/2023/8854311

**Published:** 2023-07-22

**Authors:** Jaber Hemmati, Mehdi Azizi, Babak Asghari, Mohammad Reza Arabestani

**Affiliations:** ^1^Department of Microbiology, School of Medicine, Hamadan University of Medical Sciences, Hamadan, Iran; ^2^Student Research Committee, Hamadan University of Medical Sciences, Hamadan, Iran; ^3^Department of Tissue Engineering and Biomaterials, School of Advanced Medical Sciences and Technologies, Hamadan University of Medical Sciences, Hamadan, Iran; ^4^Infectious Disease Research Center, Hamadan University of Medical Sciences, Hamadan, Iran

## Abstract

Multidrug-resistant pathogens are one of the common causes of death in burn patients and have a high risk of nosocomial infections, especially pneumonia, urinary tract infections, and cellulitis. The role of prolonged hospitalization and empirical antibiotics administration in developing multidrug-resistant pathogens is undeniable. In the early days of admitting burn patients, Gram-positive bacteria were the dominant isolates with a more sensitive antibiotic pattern. However, the emergence of Gram-negative bacteria that are more resistant later occurs. Trustworthy guideline administration in burn wards is one of the strategies to prevent multidrug-resistant pathogens. Also, a multidisciplinary therapeutic approach is an effective way to avoid antibiotic resistance that involves infectious disease specialists, pharmacists, and burn surgeons. However, the emerging resistance to conventional antimicrobial approaches (such as systemic antibiotic exposure, traditional wound dressing, and topical antibiotic ointments) among burn patients has challenged the treatment of multidrug-resistant infections, and using nanoparticles is a suitable alternative. In this review article, we will discuss different aspects of multidrug-resistant pathogens in burn wounds, emphasizing the full role of these pathogens in burn wounds and discussing the application of nanotechnology in dealing with them. Also, some advances in various types of nanomaterials, including metallic nanoparticles, liposomes, hydrogels, carbon quantum dots, and solid lipid nanoparticles in burn wound healing, will be explained.

## 1. Introduction

The skin is the body's largest organ and is considered one of the most significant barriers to body protection against external pathogens [1]. Other functions of this organ include hormonal balance, maintaining temperature and humidity, sensory perception, and burn injuries that cause loss of integrity and destruction of skin tissues [1]. Due to the self-healing properties of the skin, acute wound healing is quick, while the process of healing chronic wounds is time-consuming, infection in the affected area can prolong the wound for years [2]. Aging, obesity, diabetes, autoimmune disease, and cardiovascular problems are risk factors for chronic wounds that increase their incidences [3]. In this regard, people with diabetes are 20% more likely to develop chronic abscesses [4]. In addition, some comorbidities, such as sporotrichosis, malignant tumors, and dermatophytosis, can provide conditions for chronic wounds in burn patients [5]. Prolonged exposure to the underlying tissue of chronic wounds with the external environment and bleeding, osteomyelitis, and septicemia increase the chances of mortality among patients with chronic burn wounds [6]. Moreover, burn wounds cause other challenges, such as many mental problems, reduced quality of life, and increased medical costs in patients [7]. Besides the heavy economic burden of burn injuries, the prolonged hospitalization and employing equipment and labor are other consequences of these wounds for medical systems [7].

Burn and its resulting infections have been among the main challenges to the world's medical systems [8]. According to the reports, almost 38,000 patients were admitted to the United States burn centers and provided medical care in 2018 [9]. Despite advances in burn care over the past 50 years, burn infections are still one of the leading causes of death nowadays [10]. Over the past decade, several studies have shown that microbial infections cause death in 42 to 65 percent of burn patients [10]. Furthermore, the mortality rate in infectious burn patients is almost twice as high as in noninfectious burns [11]. The most detrimental effect of burn is the loss of a healthy skin barrier that causes the immune system to lose self-control and makes the body more susceptible to infections [12]. In addition, the more complex interaction of anti-inflammatory signals leads to the irregularities of innate and adaptive immune systems [12]. Also, increased risk in burn patients is associated with urinary and venous catheters, arterial lines, tracheal intubation, and prolonged hospitalization [13]. Antibiotic resistance emerged immediately after the discovery of the first antibiotic, which developed rapidly and is one of the main challenges for the medical community, especially in burn units [14]. There is a prediction that by 2050, multidrug-resistant (MDR) organisms will kill almost 2 million people and cost more than $3 million annually [15].

Nanotechnology has heralded a new era characterized by the development of particles composed of various types of materials, with sizes ranging from 1 to 100 nanometers [16]. In the past, nanomaterials have been introduced as vehicles for drug delivery and targeting, but at present, nanoparticles themselves can exhibit various pharmaceutical potentials [17]. Nanomedicine can improve treatments for diseases with poor prognosis, such as infection, cancer, and neurodegenerative diseases [18]. Nanotechnology has introduced innovative medical approaches for wound healing, and new therapeutic strategies based on nanosystems can be developed to fight many diseases, especially burn wound infections [19]. Current treatment modalities in burn wound infection have significant limitations, such as poor permeability, drug side effects, and enzymatic degradation, that nanotechnology could be an appropriate solution for overcoming these difficulties [20]. In addition to these advantages, nanotechnology, through the target delivery, can enhance the therapeutic profile of drugs in infected sites and reduce the risk of emerging resistant infections [21]. Therefore, the utilization of nanotechnology can lead to development in the treatment of infected burn wounds in the post-antibiotic era and faster regeneration of damaged skin [22].

## 2. Common Infections and Pathogens in Burn Patients

Infection is the most significant problem in burn wards, causing numerous treatment challenges in patients [8]. The American Burn Association (ABA) analyzed 221,519 patients in burn units between 2009 and 2018 and reported the distribution of infections that have occurred in burn patients [23]. According to the ABA report, the most common clinically relevant complications for burn patients admitted to the United States burn centers are pneumonia, urinary tract infections (UTIs), and cellulitis [23]. The highest frequency of pneumonia occurred among patients aged 50–60 years and was reported at 4.7%, while in patients over 80 years of age, the most common complication was UTI at 6.3% [23]. Other commonly reported complications included respiratory failure, wound infection, septicemia, and renal failure [23]. The duration of a mechanical ventilator can be considered a risk factor for all mentioned complications except cellulitis, wound infections, and UTIs [9, 23, 24].

The trend of nosocomial infections in burn patients is relatively predictable, and various infections and bacterial outbreaks are also associated with schedule [24]. The earliest infections occur in the skin and soft tissues that usually appear within the first week of hospitalization [25]. On the other hand, complications such as pneumonia, bloodstream infections, and UTIs typically arise in the following days of hospitalization [25]. In addition, several studies have shown that the duration of hospitalization after a burn is related to the type of bacteria isolated from the patient. A retrospective study on 125 patients admitted to a burn center confirmed the relationship between hospitalization time and the distribution of Gram-negative bacteria isolated from cultures [26]. According to this study, the significant change in the frequency of *Pseudomonas aeruginosa* was so rare in the first week of hospitalization that it accounted for only 8% of all Gram-negative isolates and reached 55% after 28 days of admissions [26]. However, *Haemophilus influenzae* rates decreased significantly from the first to the second week [26]. In addition, the emergence time of Gram-positive bacteria is much shorter than that of Gram-negative ones [24]. In this regard, on average, *Staphylococcus* spp. and *Pseudomonas* spp. are isolated from hospitalized patients three and eight days after admission, respectively [25]. Also, it has been proven that the most common bacteria causing infections within the first five days of hospitalization are *Staphylococcus aureus*, *Escherichia coli*, *H. influenzae*, and *Klebsiella pneumoniae*, while *P. aeruginosa* is the most common pathogen after five days of hospitalization [27].

## 3. Burn Wound and Multidrug-Resistant Pathogens

Multidrug-resistant pathogens are significant life-threatening agents in burn patients that have raised various concerns for healthcare systems [8]. These pathogens mainly include *P. aeruginosa*, methicillin-resistant *S. aureus* (MRSA), *Acinetobacter baumannii*, vancomycin-resistant *enterococci* (VRE), and *Stenotrophomonas maltophilia*. In addition, the outbreaks of Carbapenem-resistant *Enterobacteriaceae* (CRE) among burn patients are increasing [27–29]. In the retrospective study among burn patients between 2012 and 2017, a significant number of MDR pathogens were isolated from blood cultures, and this rate was reported between *Acinetobacter* spp. 97.5%, MRSA 97.7%, *Pseudomonas* spp. 92.2%, and CRE 31.1% [29]. In addition, the antibiogram results of wound swabs in pediatric burn patients showed that about 80% of Gram-negative isolates were MDR. Also, 100% *of E. coli* and *Klebsiella* spp., 79.2% of *P. aeruginosa,* and 69.2% of *Acinetobacter* spp. had an MDR pattern [30].

Hospitalization time is also one of the main risk factors for developing MDR infections in burn centers related to a wide range of clinical features, such as burn size and inhalation injury [24]. In this regard, 6% of the bacterial species isolated in the first week of hospitalization were MDR, while this rate increased to 44% in the fourth week [26]. In another study among more than 5,000 burn patients, a sharp increase in the proportion of Gram-negative MDR bacteria during hospitalization, and the change rate (per 1000 patient-days) of MDR pathogen from the first week to the fourth week of hospitalization ranged in the following order: CRE 0.04–0.82, extended-spectrum *β*-lactamase-producing *Enterobacteriaceae* 0.26–0.46, and fluoroquinolone-resistant *Enterobacteriaceae* 0.52–2.61 [25], as well as the MDR *Pseudomonas* spp. ranged from 0.04 to 1.85 and showed a similarly drastic change in the same hospitalization time [25]. Furthermore, the distribution of CRE on the 7th–22nd days of hospitalization significantly ranged from 16.7% to 45.0% [29].

Also, hospitalization in the intensive care unit (ICU) significantly increases in MDR infection rate [31]. A study reported that the isolation of MDR respiratory isolates in the burn intensive care unit (BICU) was about three times higher than MDR isolates in other ICUs [31]. According to the results of BICU patients, the MDR rate of *A. baumannii*, *S. aureus*, *Pseudomonas* spp., and *S. maltophilia* was 90.8%, 82.0%, 33.8%, and 21.1%, respectively [32]. Also, other risk factors for developing MDR infections in burn patients include previous exposure to antibiotics and invasive medical devices such as urine catheters and endotracheal tubes [33].

## 4. Classification of Burn Wound Infections

Delay in the epidermal layer maturation has led to eschar tissue formation, one of the main problems of burn wound infection [34]. Microbial invasion of the subcutaneous layers of injured skin can also lead to bacteremia, septicemia, and other disseminated infections [34]. The clinical diagnosis of burn infection is based on monitoring vital signs and examining the entire surface of the burn wound, especially when changing the dressing [35]. The conversion of partial thickness to deep wounds, the rapid expansion of cellulitis into healthy tissue around the injured area, evident detachment of eschars, and necrosis are among the localized symptoms of invasion in burn wound infection [34].

Traditionally, factors such as eschar formation, wound healing time, mortality rate, and specific conditions like disseminated infections, immunodeficiency, cellulitis, and impetigo are involved in classifying burn wound infections [34]. With the advent of early excision treatment, a new classification of burn wound infections associated with surgical wound infection progressed by a subcommittee of the American Burn Association's Organization and Care Committee; the following is a brief description of these classifications [34, 36].

### 4.1. Impetigo

Impetigo involves the loss of epithelium from a re-epithelialized surface, such as partial thickness or grafted burn. Burn impetigo is unrelated to hematoma formation and mechanical destruction of the graft [37]. Also, unlike other wound infections, there are no systematic infection symptoms in impetigo, such as leukocytosis, fever, and thrombocytopenia [37].

### 4.2. Cellulitis

Burn wound cellulitis is caused by the spread of infection to healthy skin and soft tissues around the burn wound [38]. This condition can be diagnosed by extending erythema to the intact skin around the burn site, which is more significant than expected [37]. Burn wound cellulitis includes at least one of the following signs: swelling or heat, pain, or local sensitivity in the affected area, progressive erythema, or edema, as well as symptoms of lymphadenitis, which spread from the injured skin along the lymphatic pathways [37]. Bacteremia and septicemia can also be symptoms of burn wound cellulitis [37, 39].

### 4.3. Burn Surgical Wound Infection

Surgical wound infections occur in incised wounds and donor tissues that are not-epithelialized [39]. The wound secretes pus which makes wound culture positive [39]. Also, appearance changes that include erythema and hyperemia of healthy skin around the wound include the features of surgical burn infection [39].

### 4.4. Unexcised Burn Wound Invasive Infection

Patients with unexcised partial-thickness or full-thickness burns are at risk of invasive infections, which are accompanied by changes in the appearance and features of the wound that can lead to darkening and/or detachment of the eschar [34]. Symptoms of invasive infection in unexcised burn wounds include inflammation, heat, swelling, erythema, and edema of the surrounding uninjured skin [34]. In addition, isolation of pathogens from blood culture in the absence of other identifiable sources of infection, signs of systemic septicemia (such as tachycardia, hypotension, oliguria, and hyperglycemia), and evidence of microbial invasion of the underlying tissues on histopathological examination are other symptoms of this type of burn wound infections [34, 37]. The most common pathogens that are related to unexcised burn invasive infection include *P. aeruginosa*, *S. aureus*, *A. baumannii*, and *Enterobacteriaceae* [39, 40].

## 5. Wound Healing Process

**Figure 1:**
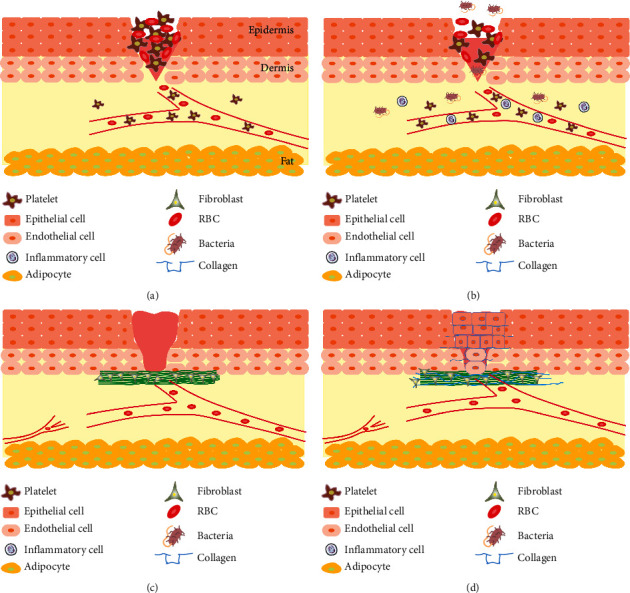
Representation of burn wound healing process: (a) hemostasis, (b) inflammatory, (c) proliferation, and (d) regeneration stages.

The restoration wound process is very complicated and consists of 4 stages that usually overlap and are affected by each other [41]. These stages include hemostasis, inflammatory, proliferation, and regeneration; various cells and biomolecules are involved in each stage (Figure 1) [42].

### 5.1. Hemostasis Stage

This stage, also known as exudative and coagulative, begins immediately after the injury, preventing vascular bleeding [43, 44]. Also, cell signaling through the secretion of cytokines and multiple growth factors causes the migration of fibroblasts and endothelial, immune, and progenitor cells to the injured site [45]. Furthermore, angiogenesis, vasoconstriction, and clot formation contribute to the goals of hemostasis in this stage, which also affect other subsequent stages of wound healing [46].

### 5.2. Inflammatory Stage

This phase usually lasts up to 72 hours after the injury [47], and platelets stimulate the release of inflammatory mediators from cells such as mast cells and basophils, leading to inflammation, heat, and vasodilation [48]. In addition, platelets cause the absorption of immune cells to the affected area by releasing several growth factors involved in cleaning pathogens and debridement [48].

### 5.3. Proliferation Stage

This stage begins on the fourth day of the healing process and can take up to 21 days, depending on the patient's safety level, size, and type of wound [49]. In addition to angiogenesis, cell proliferation and elastin production occur at this stage, and tissue granulation replaces the clot formed at the affected site [50, 51]. Furthermore, wound contraction is performed by differentiating fibroblasts into myofibroblasts, leading to a boundary between the healthy and damaged areas [46].

### 5.4. Regeneration Stage

This step is the balance between the synthesis and destruction of damaged tissue, and it can last one year or even more [2]. In addition, collagen fibers involve in scar formation, which causes wound closure and strengthens the skin at the injured site [41]. Besides reinforcing the connective tissue and epithelial layer in this stage [52], the apoptosis process destroys the unnecessary cells at the regenerated site [53].

## 6. Prevention of Multidrug-Resistant Infections

The significant impact of infection prevention approaches on the recovery and survival of burn patients is undeniable [24]. Burn patients are at increased risk of nosocomial infections due to prolonged hospitalization and frequent invasive procedures [10]. Therefore, strategies such as disinfecting the hospital environment, hand hygiene, and patient isolation are among the critical control approaches to help minimize nosocomial infection in burn wards [54]. Numerous studies have confirmed that these control approaches effectively prevent the development of MDR pathogens in burn patients [10, 24, 55]. However, using shared water resources such as hydrotherapy rooms among burn patients makes difficult to implement a precise infection control strategy. In this regard, in the Swiss burn center, the persistent outbreak of MDR *P. aeruginosa* occurred in 23 groups of patients who had been infected with the same bacterial genotype for more than three years. Interestingly, the infection outbreak was restricted by environmental cleaning measures and the disinfection of the hydrotherapy rooms [56].

Several techniques should be used in the burn wards for monitoring of MDR pathogens, especially VRE, MRSA, and CRE [28]. Also, the high incidence of MDR bacteria in burn units requires fully considering the cost, time, risks, benefits, and flaws of different screening methods [10]. For example, three days per week of endotracheal aspirate cultures in patients with inhalation injuries is recommended to identify MDR strains in ventilator-associated pneumonia (VAP) [57]. However, further information is needed to determine whether monitoring cultures should be carried out routinely in burn wards.

Intravascular catheters are one of the biggest challenges in burn patients, and according to the guidelines of the Infectious Diseases Society of America (IDSA) and the Society for Healthcare Epidemiology of America (SHEA), most burn centers do not regularly replace these catheters [58, 59]. However, there are conflicting results in a review of various studies. In this regard, the study found that the rate of catheter-related bloodstream infections in patients who had their catheter replaced every four days increased compared to the 3-day replacement [60]. Nevertheless, in another study, increasing the duration of catheter use from 48 hours to 72 hours did not increase the catheter-related infection rate [61]. Based on these limited data, some burn wards change catheters every 72 hours [24]. Therefore, more investigations, including controlled trials, are needed to understand the duration of intravascular catheter replacement in burn patients.

In addition, early excision and graft of burnt tissue can have a significant effect on reducing the prevalence of infections and as well as mortality in burn patients [35]. Removal of incurable and necrotic tissues should also be part of burn patients' routine and necessary care procedures [62]. Moreover, it has been shown that administration of topical antimicrobial agents (such as silver sulfadiazine and mafenide), combined with excision, can reduce the incidence of septicemia induced by burn wound infections [63].

There is also contradictory information on systemic antibiotic prophylaxis to control infections in burn patients. In this regard, in a study, the systemic antibiotic prophylaxis in nonsurgical patients was evaluated in three trials (119 participants), and there was no evidence of an effect on rates of burn wound infection [64]. However, the results of this study revealed that systemic antibiotics (trimethoprim‐sulfamethoxazole) significantly decreased pneumonia rates in patients with burn wounds [64]. The data of another study indicated that the prophylactic antibiotics (ampicillin/sulbactam and first-generation cephalosporin) reduced mortality in mechanically ventilated patients with severe burns but not in those who do not receive mechanical ventilation [65]. In addition, another study supports the routine usage of antibiotic prophylaxis in patients with inhalational burns and developing pneumonia [66]. Also, another study showed that resistance to the antibiotic administered for prophylaxis in burn patients significantly increased [67]. Furthermore, another study considers the importance of the grading system in the effectiveness of systemic antibiotic prophylaxis in burn patients [68]. However, the benefit of prophylaxis in patients with burn wounds requires more information, while the International Society for Burn Injury (ISBI) has not yet recommended systemic antibiotic prophylaxis for burn injury [69].

## 7. Diagnosis of Multidrug-Resistant Pathogens

Due to the high prevalence of MDR bacterial infections among burn patients, early diagnosis can play an influential role in reducing the mortality of these patients [10]. However, the main difficulty in diagnosing MDR bacterial infection in burn patients is the distinction between colonization and infection [10]. The colonization of the respiratory tract and endotracheal tubes after severe burns in patients with prolonged ventilation indicates the precedence of colonization over infection [24]. Another example is the colonization of urinary bacteria in patients who have long used urinary catheters [24]. Unfortunately, most patients with severe burns are in critical condition and do not provide relevant clinical information to diagnose infection [70].

Acute respiratory distress syndrome and inhalation injury are some of the problems that make it challenging to diagnose VAP in burn patients [10]. In addition, the influential role of bronchoscopy in diagnosing pneumonia in burn patients was also approved. According to the National Burn Repository data, patients who underwent bronchoscopy were 18% less likely to die from pneumonia than those who did not have bronchoscopy [71]. Also, the identification of MDR bacteria causing VAP with bronchoscopy may help determine the course of antimicrobial therapy and can more effectively treat pneumonia in burn patients [72].

The distinction between noninfectious burn erythema and other similar skin infections in burn patients, such as invasive burn wound infection and burn cellulite, is sophisticated [34]. In invasive burn wound infections, the gold standard for diagnosing is still tissue biopsy and histopathology, but these approaches are often not carried out due to being costly and labor-intensive [34]. Also, in several studies, surface swab and tissue biopsy culture has been proposed as suitable methods for diagnosing skin infection in burn patients [24, 34, 73]. In addition, several studies suggested swab cultures for regular monitoring of burn wound infections with insufficient skin tissue for biopsy [10, 24, 34]. Furthermore, swab culture and biopsy are recommended in burn patients with systemic infection symptoms because they increase the chance of identifying the source of infection, especially MDR bacteria [74]. However, there are some evidences to suggest that a biopsy or swab may not be able to detect all bacteria involved in burn wound infection [24]. Besides being costly and labor-intensive, the need for surgical preparation, invasive sampling, and patient noncompliance are among disadvantages of the biopsy method for monitoring of burn wound infection [75]. Swab culturing has little use in the identification of bacterial infection in burn patients because it is time-consuming, requires frequent sample, is contaminated with bacterial skin flora, is low-sensitive, and results in deep skin infections [76–78].

According to the ABA Consensus Conference, the definition of infection and sepsis in burn patients includes at least three clinical symptoms: hypo/hyperthermia, hyperglycemia, tachypnea, tachycardia, and thrombocytopenia [38]. The diagnosis of sepsis in burn patients is also complicated because systemic symptoms, such as fever, leukocytosis, and hypotension, may have occurred in noninfectious burn patients [74]. Moreover, clinical trials have not shown successful guidance in diagnosing septicemia in burn patients [38]. However, the determination of procalcitonin level in the diagnosis of burn sepsis seems promising [79]. In this regard, a study introduced an antibiotic algorithm based on procalcitonin level that indicates sepsis in burn patients could be treated on average five days earlier [80].

## 8. Antibiotics Therapy of Multidrug-Resistant Pathogens

When deciding whether to treat a burn patient with suspected or confirmed bacterial MDR infection, some specific issues including timely eradicating the infection sources must be considered [81]. In this regard, removing eschar in deep wound infections and burn wound cellulitis usually leads to rapid limitation of the infection [81]. Also, removing contaminated catheters, especially those colonized with biofilm-forming pathogens, effectively improves the treatment outcomes in burn patients [82].

One notable point in treating burn infections is optimizing empirical antimicrobial therapy by analyzing antibiogram data collected from all burn wards [83]. However, guidelines such as the antimicrobial stewardship program (ASP) are recommended to reduce patients' antibiotic exposure and the prevalence of bacterial MDR infection [84]. In this regard, a study has proven that the administration of ASP could decrease drug resistance without much adverse effect on the patient [85]. Also, the ISBI suggested that a specific ASP be developed in burn centers to enable research on microbial resistance in burn patients [69].

Prescribing antibiotics for severe burns becomes more difficult due to the hyperdynamic status of patients, including high renal clearance, which causes patients to need more significant amounts of conventional antibiotics [84, 85]. There are also conflicts about the effect of antibiotic combination therapy on the development of MDR bacteria, as the main challenges in treating burn patients, and require further studies [24, 86]. In addition, the exact role of some antimicrobial agents, such as cephalosporin/beta-lactamase inhibitor compound combinations, novel cephalosporins, and long-effective anti-MRSA antibiotics, is yet to be determined in burn patients [24]. However, the appointment of a pharmacist, infectious disease specialists, and burn surgeons in the multidisciplinary burn team is necessary to effectively treat infectious patients [87].  Table 1 lists the approved and controversial strategies for controlling MDR pathogens in burn injury.

## 9. Nanostructures as a Suitable Platform for Overcoming the MDR Pathogens in Burn Wounds

MDR pathogens are primary challenges in burn patients induced by high systemic antibiotic exposure [8]. Also, the topical administration of antibiotics could not be an effective wound-healing strategy due to the skin barrier dysfunction following a burn [88]. Nanostructures as advanced drug delivery systems can reduce systemic drug dosage and increase the potential for topical administration, which could be developed as suitable candidates for treating burn wound infection [89]. However, the kinetics and dynamics of nanostructures depend on several factors, such as the degree of injured skin, the presence of infection in the burn wound, and nanoparticle properties (such as size, type, and half-life) [90]. In addition, an ideal nanoparticle for topical application in burn wounds must have essential features such as nonimmunologic, nontoxicity, biodegradability, and appropriate release profile [91, 92]. Moreover, the source of nanoparticles (organic or inorganic compounds) has an effective role in reducing their side effects that should be considered for development in wound healing [19].  Table 2 lists some of the advances of nanomaterials for overcoming MDR pathogens in burn wounds.

**Figure 2:**
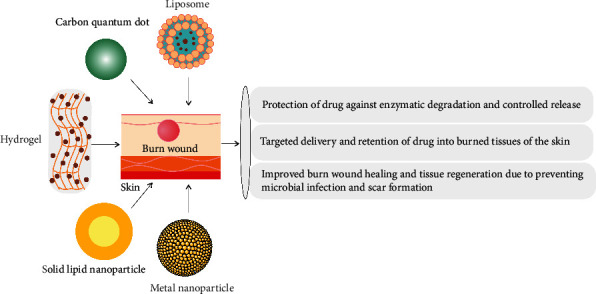
Schematic representation of nanomaterials used for burn wound healing. Metallic nanoparticles, liposome, hydrogel, carbon quantum dot, and solid lipid nanoparticles.

The ineffectiveness of traditional wound therapies increases the need for the development and discovery of new strategies in wound healing [111]. Recently, nanotechnology-based approaches have announced an effective wound‐healing platform with a high potential for curing burn wounds [111] (Figure 2). Also, the antimicrobial activity of nanocarriers, as well as their high ability to deliver antimicrobial agents, has been applied to increasing wound closure [112]. In addition, nanostructures can be administered as burn wound dressing by mimicking the skin's extracellular matrix and encapsulating active ingredients [112].

### 9.1. Metallic Nanoparticles

**Figure 3:**
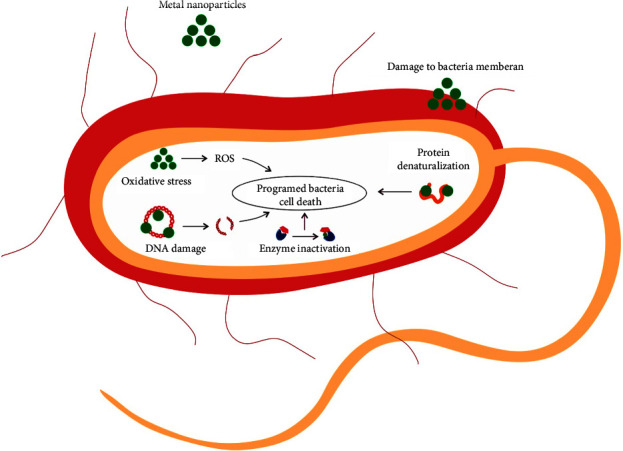
Mechanisms of antibacterial action of MNPs. MNPs promote programmed bacterial cell death through enzyme inactivation, protein denaturalization, DNA damage, ROS generation, and cell membrane damage. MNPs: Metallic nanoparticles; ROS: reactive oxygen species.

Recently, metallic nanoparticles (MNPs) such as silver, zinc oxide, gold, titanium dioxide, and copper attracted special consideration because of their distinct properties [113]. The large surface area-to-volume ratio yields numerous chemically active sites in MNPs, which improves their therapeutic and pharmaceutical efficacy [114]. Also, MNPs could increase the accumulation of antimicrobial agents at the infected sites through magnetic-field-controlled release [115]. In the last decade, the use of MNPs to overcome MDR infections has expanded [113], and the high potency of these nanoparticles has been proven to successfully prevent and eradicate of wound infections [116–118]. Metal ions have extensive antimicrobial properties such as cell membrane damage, production of reactive oxygen species (ROS), targeting functional groups of metabolites, disruption of electron transfer chains, protein dysfunction, destruction of DNA, and repair systems (Figure 3) [119–121]. Silver NPs are one of the well-known MNPs [19, 122] that possess properties such as catalytic activity, chemical stability, low cost, and broad-spectrum resistance to numerous pathogens, which make them an appropriate candidate for combating resistant infections and also burn wound healing [113, 123]. Other MNPs with antimicrobial properties for wound healing are zinc oxide NPs, which could regenerate damaged skin by reducing necrosis, controlling infection, collagen fiber deposition, and re-epithelialization [113, 124]. Despite the high cost, the outstanding features of gold NPs, including antioxidant, anti-inflammatory, antimicrobial action, and particularly enhancing scaffold properties, could increase their application in wound healing [113, 125]. Furthermore, titanium dioxide NPs can effectively control skin infections caused by Gram-positive and -negative bacteria that are approved as a wound healer agent [123]. Like mentioned MNPs, copper NPs showed potent microcidal activities [113]. Also, these nanoparticles could improve the wound healing process by contributing to collagen formation, enhancing immunity, and angiogenesis [113]. However, the affordability, mechanism of action, synthesis methods, and cytotoxicity are among the factors that should be considered for developing MNPs in burn wound healing [113, 126, 127].

### 9.2. Liposome

Liposome nanoparticles of natural origin, usually phospholipids, are among the main high-performance nanocarriers in the drug delivery system [128]. Low toxicity, biocompatibility, stability, sustained drug release, prolonged systemic circulation, and long residual time in the targeted site are some of the characteristics of liposomes that can make them excellent candidates for eradicating resistance pathogens in burn wounds [129, 130]. Also, the delivery of antimicrobial drugs to target sites and bacteria is among the significant functional aspects of liposomes [130–132]. In addition, liposome effectiveness against resistant wound infection could be improved by changing composition and using different materials in the liposomal formulation (Table 2) [133]. In this regard, the study showed that the epidermal growth factor-containing liposome formulation has an effective role in the treatment of burn wounds [134]. Also, the synthesized formulation increased the epidermal thickness, fibroblast, and collagen fibers at the injured site, which could be a promising strategy for wound healing [134]. Furthermore, the results of another study indicated that liposomes, as an effective vehicle for antimicrobial agents, could enhance local drug concentration at the site of injury [135]. Moreover, the liposome-loaded scaffolds were proven to have therapeutic effects on skin regeneration and could be proposed as a potent agent for burn wound healing [136].

### 9.3. Hydrogel

Hydrogels are three-dimensional soft polymers composed of nanometer-scale particles with numerous properties, making them the ideal nanostructures for wound healing and infection control [137]. These features include high water content, optimal stability, and suitable chemical and mechanical properties [138]. One of the essential strategies to tackle MDR pathogens is to increase the bioavailability of antimicrobial agents in the infection sites. Recent advances in the development of hydrogels have achieved not only this factor but also can reduce the side effects of drugs [139–142]. The encapsulation and conjugation of biocidal agents, such as conventional antibiotics [107, 143–145] and antimicrobial peptides [146–149], into hydrogels have a significant role in developing these nanostructures. In this regard, in several studies, hydrogels have been used for antibiotic delivery to infection sites with desirable treatment outcomes [107, 150–152].

Furthermore, it has been proven that MNPs, in combination with hydrogels, have greater therapeutic efficacy in burn wounds [153, 154]. In this regard, the hydrogel system containing silver NPs with significant microbicidal effects on *S. aureus* and *E. coli* was introduced as a suitable candidate for burn wound healing [155]. Also, a hydrogel-embedded silver-binding peptide is a promising injectable strategy for wound dressing, which conquers the toxicity of silver by controlling release patterns [156]. In addition, incorporating silver sulfadiazine with hydrogel can promote angiogenesis, re-epithelialization, and collagenases in injured tissues [157]. In several studies, the physical, chemical, and biological properties of various nanocomposite hydrogels embedded with silver NPs have been investigated. In this regard, the study [158] showed that the encapsulation of silver nanoparticles within lignin-based hydrogels could improve their antibacterial properties towards both *S. aureus* and *E. coli* [158]. Also, this study approved the biocompatibility, mechanically stability, and rheological properties of hydrogel-silver nanocomposites, which can be a promising approach for wound healing [158]. In another study, the collagen-silver NPs hydrogels displayed remarkable properties, including biocompatibility with fibroblasts, anti-inflammatory, and broad-spectrum antimicrobial ability [159]. In addition, silver NPs impregnated chitosan, polyvinyl alcohol, and polyethylene glycol hydrogels were proposed as biocomposite dressings, each of which has various attributes that should be considered in the development of hydrogels for burn wound healing [160, 161].

### 9.4. Carbon Quantum Dot

**Figure 4:**
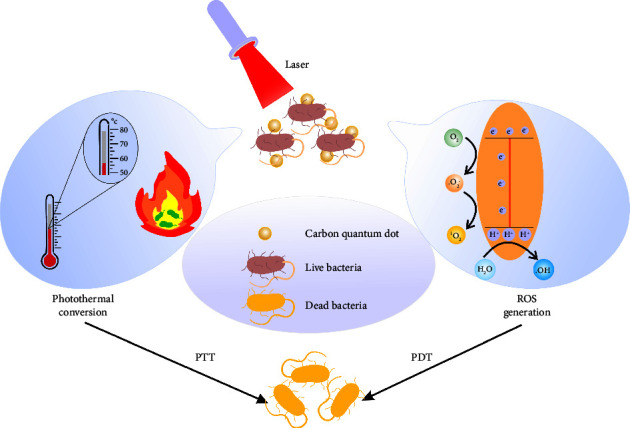
Representation of the antibacterial mechanism of carbon quantum dots. In PTT and PDT, the temperature and reactive oxygen species are activated by light radiation to kill bacterial cells, respectively. PTT: photothermal therapy; PDT: photodynamic therapy.

Carbon quantum dots (CQDs) are carbon-based nanoparticles with a diameter size of fewer than 10 nanometers [162]. Outstanding properties such as high chemical stability, significant water solubility, excellent biocompatibility, low toxicity, and exceptional photoelectric capacity make them appropriate for antibacterial applications [163]. Generally, CQDs and other nanostructures that can absorb light radiation could be used for photothermal and photodynamic therapies, which are excellent strategies to overcome MDR pathogens. In these therapeutic methods, light radiation destroys a bacterial cell's membrane, proteins, and DNA. Enhancing the production of the temperature and reactive oxygen species by the emitted waves leads to the death of bacteria in photothermal and photodynamic therapy, respectively [164–166]. Besides killing bacteria, heat generation in photothermal therapy could significantly reduce the risk of developing resistance mechanism (Figure 4) [167, 168].

### 9.5. Solid Lipid Nanoparticle

Solid lipid nanoparticles (SLNs) are lipid-based nanoparticles that are widely used in drug delivery systems. Besides the advantages of liposomes, SLNs have a higher ability to load hydrophobic and hydrophilic materials that can be a suitable alternative for liposomes [169–171]. SLNs could be developed to deal with MDR pathogens that delay in tissue regeneration following burn wound [172]. Also, SLNs significantly improve the therapeutic effects in infected wounds through different mechanism, including reducing the activity of bacterial efflux pumps [173], inhibiting enzymatic degradation of antimicrobial agents, and increasing drug accumulation at infected sites [169]. In this regard, the result of the study showed that ampicillin-loaded SLNs with significant antibacterial efficacy could increase the rate of burn wound healing [174]. In addition, it has been proven that methylene blue-loaded SLNs accelerate the healing process and could be a suitable strategy for treating burn wounds [175]. Furthermore, in another study, silver sulfadiazine SLNs were introduced as appropriate candidates for burn wound dressing with good bioadhesive behavior [176].

## 10. Conclusion

Due to the prolonged hospitalization and high antibiotic exposure, patients with burn wounds are prone to the emergence of MDR pathogens. Also, the high prevalence of MDR in burn wards can be attributed to several patients' risk factors, including high colony formation, hyperdynamic status, surgical treatment, and immunodeficiency conditions. Preventing the outbreak of MDR bacteria in this population requires a multistep approach, including hand hygiene, antimicrobial care, operation optimization, careful use of medical equipment, and environmental controls. It strongly recommends the involvement of infectious disease specialists, burn surgeons, and pharmacists in routine therapeutic measures of burn patients. Given the many social and economic consequences of burn wounds and also challenges in treating of resistant infections caused by these wounds, efforts to develop new and efficient wound-healing strategies are essential. Nanotechnology is an exciting emerging field with multiple applications in medicine and can be a promising approach to wound healing based on its different properties, including antimicrobial activity, reduced toxicity, controlled release profiles, and similarity to the extracellular matrix. Also, nanoparticles could deliver antimicrobial agents and growth factors to the injured site, leading to improved healing outcomes. Furthermore, nanostructured scaffolds could be developed as an ideal wound dressing due to their potential to promote skin regeneration and management of burn injuries. Therefore, the application of nanotechnology in wound care shows great promise for reducing the treatment challenges in burn patients.

## Figures and Tables

**Table 1:** Prevention, diagnosis, and treatment strategies of multidrug-resistant pathogens in burn patients.

Approved strategies	(1) Hand hygiene and environmental disinfection protocols
	(2) Isolation of infectious patients caused multidrug-resistant pathogen
	(3) Use of antimicrobial stewardship program
	(4) Graft and excision burn tissue
	(5) Administration of topical antimicrobial agents
	(6) Development of a local antibiogram pattern
	(7) Engagement of infectious disease specialists, burn surgeons, and pharmacists
	(8) Bronchoscopy for help in the diagnosis of ventilator-associated pneumonia in inhalational burn injury
	(9) Wound swabs or biopsy cultures for diagnosis of burn infection
	(10) Removing contaminated catheters with biofilm-forming pathogens

Controversial strategies	(1) Systemic antibiotics prophylaxis and combination therapy for control infections
	(2) Routine central venous catheter replacement
	(3) Antibiotic therapy based on procalcitonin level in burn sepsis

**Table 2:** The advances of nanostructures for the treatment of MDR pathogens in burn wounds.

Nanoparticle	MDR pathogen	Effect	References
Liposome	Methicillin-resistant *Staphylococcus aureus* (MRSA)	Increased drug concentration in infected tissues	[93–96]
	Vancomycin-resistant* enterococci* (VRE)	Enhanced antimicrobial activity	[97]

Solid lipid nanoparticle	Carbapenem-resistant *Enterobacteriaceae* (CRE)	Prolonged antibacterial activity	[98, 99]

Metallic nanoparticle	Methicillin-resistant *Staphylococcus aureus* (MRSA)	Enhanced therapeutic profile	[100–102]
	Vancomycin-resistant *enterococci* (VRE)	Enhanced bactericidal activity	[103, 104]
	Carbapenem-resistant *Acinetobacter baumannii* (CRAB)	Synergistic effects in combination with antibiotics	[105, 106]

Hydrogel	Methicillin-resistant *Staphylococcus aureus* (MRSA)	Improved therapeutic profile of the encapsulated drug	[107]

Carbon quantum dot	Methicillin-resistant *Staphylococcus aureus* (MRSA)	Photodynamic therapy	[108]
	Vancomycin-resistant* enterococci* (VRE)		
	Methicillin-resistant* Staphylococcus aureus* (MRSA)	DNA and membrane damage caused by oxidative stress	[109]
	Methicillin-resistant *Staphylococcus aureus* (MRSA)	Photothermal therapy	[110]
	Carbapenem-resistant *Escherichia coli*		

## Data Availability

The datasets used and/or analyzed during the current study are available from the corresponding author on reasonable request.

## References

[1] Dąbrowska A., Spano F., Derler S., Adlhart C., Spencer N. D., Rossi R. M. (2018). The relationship between skin function, barrier properties, and body‐dependent factors. *Skin Research and Technology*.

[2] Hoversten K. P., Kiemele L. J., Stolp A. M., Takahashi P. Y., Verdoorn B. P. (2020). Prevention, diagnosis, and management of chronic wounds in older adults. *Mayo Clinic Proceedings*.

[3] Oliveira A., Simões S., Ascenso A., Reis C. P. (2020). Therapeutic advances in wound healing. *Journal of Dermatological Treatment*.

[4] Spampinato S. F., Caruso G. I., De Pasquale R., Sortino M. A., Merlo S. (2020). The treatment of impaired wound healing in diabetes: looking among old drugs. *Pharmaceuticals*.

[5] Wang M., Huang X., Zheng H. (2021). Nanomaterials applied in wound healing: mechanisms, limitations and perspectives. *Journal of Controlled Release*.

[6] Kushwaha A., Goswami L., Kim B. S. (2022). Nanomaterial-based therapy for wound healing. *Nanomaterials*.

[7] Gainza G., Villullas S., Pedraz J. L., Hernandez R. M., Igartua M. (2015). Advances in drug delivery systems (DDSs) to release growth factors for wound healing and skin regeneration. *Nanomedicine: Nanotechnology, Biology and Medicine*.

[8] Branski L. K., Al-Mousawi A., Rivero H., Jeschke M. G., Sanford A. P., Herndon D. N. (2009). Emerging infections in burns. *Surgical Infections*.

[9] American Burn Association (2012). *National Burn Repository*.

[10] Vinaik R., Barayan D., Shahrokhi S., Jeschke M. G. (2019). Management and prevention of drug resistant infections in burn patients. *Expert Review of Anti-infective Therapy*.

[11] Alp E., Coruh A., Gunay G. K., Yontar Y., Doganay M. (2012). Risk factors for nosocomial infection and mortality in burn patients: 10 years of experience at a university hospital. *Journal of Burn Care and Research*.

[12] Neely C. J., Kartchner L. B., Mendoza A. E. (2014). Flagellin treatment prevents increased susceptibility to systemic bacterial infection after injury by inhibiting anti-inflammatory IL-10+ IL-12-neutrophil polarization. *PLoS One*.

[13] Schultz L., Walker S. A., Elligsen M. (2013). Identification of predictors of early infection in acute burn patients. *Burns*.

[14] Ventola C. L. (2015). The antibiotic resistance crisis: part 1: causes and threats. *P and T: A Peer-Reviewed Journal for Formulary Management*.

[15] Polinarski M. A., Beal A. L., Silva F. E. (2021). New perspectives of using chitosan, silver, and chitosan–silver nanoparticles against multidrug‐resistant bacteria. *Particle & Particle Systems Characterization*.

[16] Bhattacharya D., Ghosh B., Mukhopadhyay M. (2019). Development of nanotechnology for advancement and application in wound healing: a review. *IET Nanobiotechnology*.

[17] Hissae Yassue-Cordeiro P., Henrique Zandonai C., Pereira Genesi B. (2019). Development of chitosan/silver sulfadiazine/zeolite composite films for wound dressing. *Pharmaceutics*.

[18] Daliu P., Santini A., Novellino E. (2019). From pharmaceuticals to nutraceuticals: bridging disease prevention and management. *Expert Review of Clinical Pharmacology*.

[19] Souto E. B., Ribeiro A. F., Ferreira M. I. (2020). New nanotechnologies for the treatment and repair of skin burns infections. *International Journal of Molecular Sciences*.

[20] Hussain Z., Thu H. E., Rawas-Qalaji M., Naseem M., Khan S., Sohail M. (2022). Recent developments and advanced strategies for promoting burn wound healing. *Journal of Drug Delivery Science and Technology*.

[21] Whittam A. J., Maan Z. N., Duscher D. (2016). Challenges and opportunities in drug delivery for wound healing. *Advances in Wound Care*.

[22] Tocco I., Zavan B., Bassetto F., Vindigni V. (2012). Nanotechnology-based therapies for skin wound regeneration. *Journal of Nanomaterials*.

[23] American Burn Association (2019). *National Burn Repository 2019 Report Dataset Version 14.0*.

[24] Lachiewicz A. M., Hauck C. G., Weber D. J., Cairns B. A., Van Duin D. (2017). Bacterial infections after burn injuries: impact of multidrug resistance. *Clinical Infectious Diseases*.

[25] van Duin D., Strassle P. D., DiBiase L. M. (2016). Timeline of health care–associated infections and pathogens after burn injuries. *American Journal of Infection Control*.

[26] Wanis M., Walker S. A., Daneman N. (2016). Impact of hospital length of stay on the distribution of Gram negative bacteria and likelihood of isolating a resistant organism in a Canadian burn center. *Burns*.

[27] D’Abbondanza J. A., Shahrokhi S. (2021). Burn infection and burn sepsis. *Surgical Infections*.

[28] Kanamori H., Parobek C. M., Juliano J. J. (2017). A prolonged outbreak of KPC-3-producing *Enterobacter cloacae* and *Klebsiella pneumoniae* driven by multiple mechanisms of resistance transmission at a large academic burn center. *Antimicrobial Agents and Chemotherapy*.

[29] Park J. J., Seo Y. B., Choi Y. K., Kym D., Lee J. (2020). Changes in the prevalence of causative pathogens isolated from severe burn patients from 2012 to 2017. *Burns*.

[30] Kabanangi F., Joachim A., Nkuwi E. J., Manyahi J., Moyo S., Majigo M. (2021). High level of multidrug-resistant gram-negative pathogens causing burn wound infections in hospitalized children in dar es salaam, Tanzania. *International journal of microbiology*.

[31] Lachiewicz A. M., van Duin D., DiBiase L. M. (2015). Rates of hospital-associated respiratory infections and associated pathogens in a regional burn center. *Infection Control & Hospital Epidemiology*.

[32] Weber D. J., van Duin D., DiBiase L. M. (2014). Healthcare-associated infections among patients in a large burn intensive care unit: incidence and pathogens, 2008–2012. *Infection Control & Hospital Epidemiology*.

[33] Cavalcante R. D. S., Canet P., Fortaleza C. M. C. B. (2014). Risk factors for the acquisition of imipenem-resistant Acinetobacter baumannii in a burn unit: an appraisal of the effect of colonization pressure. *Scandinavian Journal of Infectious Diseases*.

[34] Church D., Elsayed S., Reid O., Winston B., Lindsay R. (2006). Burn wound infections. *Clinical Microbiology Reviews*.

[35] Noor A., Afzal A., Masood R. (2022). Dressings for burn wound: a review. *Journal of Materials Science*.

[36] Barret J. P., Herndon D. N. (2003). Effects of burn wound excision on bacterial colonization and invasion. *Plastic and Reconstructive Surgery*.

[37] Cambiaso-Daniel J., Gallagher J. J., Norbury W. B., Finnerty C. C., Herndon D. N., Culnan D. M. (2018). Treatment of infection in burn patients. *Total Burn Care*.

[38] Greenhalgh D. G., Saffle J. R., Holmes J. H. (2007). American Burn Association consensus conference to define sepsis and infection in burns. *Journal of Burn Care and Research*.

[39] Lazarescu A.-L., Grosu-Bularda A., Andrei M.-C. (2021). Burn infections characteristics: a review. *Romanian JouRnal of medical PRactice*.

[40] Silla R., Fong J., Wright J., Wood F. (2006). Infection in acute burn wounds following the Bali bombings: a comparative prospective audit. *Burns*.

[41] Gurtner G. C., Werner S., Barrandon Y., Longaker M. T. (2008). Wound repair and regeneration. *Nature*.

[42] Victor P., Sarada D., Ramkumar K. M. (2020). Pharmacological activation of Nrf2 promotes wound healing. *European Journal of Pharmacology*.

[43] Rodrigues M., Kosaric N., Bonham C. A., Gurtner G. C. (2019). Wound healing: a cellular perspective. *Physiological Reviews*.

[44] Matter M. T., Probst S., Läuchli S., Herrmann I. K. (2020). Uniting drug and delivery: metal oxide hybrid nanotherapeutics for skin wound care. *Pharmaceutics*.

[45] Liang J., Cui L., Li J., Guan S., Zhang K., Li J. (2021). Aloe vera: a medicinal plant used in skin wound healing. *Tissue Engineering Part B Reviews*.

[46] Sharifi S., Hajipour M. J., Gould L., Mahmoudi M. (2020). Nanomedicine in healing chronic wounds: opportunities and challenges. *Molecular Pharmaceutics*.

[47] Cañedo-Dorantes L., Cañedo-Ayala M. (2019). Skin acute wound healing: a comprehensive review. *International Journal of Inflammation*.

[48] Kratofil R. M., Kubes P., Deniset J. F. (2017). Monocyte conversion during inflammation and injury. *Arteriosclerosis, Thrombosis, and Vascular Biology*.

[49] Landén N. X., Li D., Ståhle M. (2016). Transition from inflammation to proliferation: a critical step during wound healing. *Cellular and Molecular Life Sciences*.

[50] Pastar I., Stojadinovic O., Yin N. C. (2014). Epithelialization in wound healing: a comprehensive review. *Advances in Wound Care*.

[51] Debone H. S., Lopes P. S., Severino P., Yoshida C. M. P., Souto E. B., da Silva C. F. (2019). Chitosan/Copaiba oleoresin films for would dressing application. *International Journal of Pharmaceutics*.

[52] Xue M., Jackson C. J. (2015). Extracellular matrix reorganization during wound healing and its impact on abnormal scarring. *Advances in Wound Care*.

[53] Mofazzal Jahromi M. A., Sahandi Zangabad P., Moosavi Basri S. M. (2018). Nanomedicine and advanced technologies for burns: preventing infection and facilitating wound healing. *Advanced Drug Delivery Reviews*.

[54] Yokoe D., Anderson D., Berenholtz S. (2014). A compendium of strategies to prevent healthcare-associated infections in acute care hospitals: 2014 updates. *Infection Control and Hospital Epidemiology*.

[55] van Duin D., Jones S. W., Dibiase L. (2014). Reduction in central line–associated bloodstream infections in patients with burns. *Infection Control & Hospital Epidemiology*.

[56] Tissot F., Blanc D., Basset P. (2016). New genotyping method discovers sustained nosocomial *Pseudomonas aeruginosa* outbreak in an intensive care burn unit. *Journal of Hospital Infection*.

[57] Brusselaers N., Logie D., Vogelaers D., Monstrey S., Blot S. (2012). Burns, inhalation injury and ventilator-associated pneumonia: value of routine surveillance cultures. *Burns*.

[58] O’Grady N. P., Alexander M., Burns L. A. (2011). Summary of recommendations: guidelines for the prevention of intravascular catheter-related infections. *Clinical Infectious Diseases*.

[59] Sood G., Heath D., Adams K. (2013). Survey of central line–associated bloodstream infection prevention practices across American Burn Association–certified adult burn units. *Infection Control & Hospital Epidemiology*.

[60] King B., Schulman C. I., Pepe A., Pappas P., Varas R., Namias N. (2007). Timing of central venous catheter exchange and frequency of bacteremia in burn patients. *Journal of Burn Care and Research*.

[61] Kagan R. J., Neely A. N., Rieman M. T. (2014). A performance improvement initiative to determine the impact of increasing the time interval between changing centrally placed intravascular catheters. *Journal of Burn Care and Research*.

[62] Norbury W., Herndon D., Tanksley J., Jeschke M., Finnerty C. (2016). Infection in burns. *Surgical Infections*.

[63] Brown T. P. L. H., Cancio L. C., McManus A. T., Mason A. D. (2004). Survival benefit conferred by topical antimicrobial preparations in burn patients: a historical perspective. *The Journal of Trauma, Injury, Infection, and Critical Care*.

[64] Barajas‐Nava L. A., López‐Alcalde J., i Figuls M. R., Solà I., Cosp X. B. (2013). Antibiotic prophylaxis for preventing burn wound infection. *Cochrane Database of Systematic Reviews*.

[65] Tagami T., Matsui H., Fushimi K., Yasunaga H. (2016). Prophylactic antibiotics may improve outcome in patients with severe burns requiring mechanical ventilation: propensity score analysis of a Japanese nationwide database. *Clinical Infectious Diseases*.

[66] Muthukumar V., Arumugam P. K., Bamal R. (2020). Role of systemic antibiotic prophylaxis in acute burns: a retrospective analysis from a tertiary care center. *Burns*.

[67] Avni T., Levcovich A., Ad-El D. D., Leibovici L., Paul M. (2010). Prophylactic antibiotics for burns patients: systematic review and meta-analysis. *BMJ*.

[68] Ramos G. E. (2018). Antibiotic prophylaxis in burn patients: a review of current trends and recommendations for treatment. *Journal of Infectiology*.

[69] Ahuja R. B., Ahuja R. B., Gibran N. (2016). ISBI practice guidelines for burn care. *Burns*.

[70] D’Avignon L. C., Chung K. K., Saffle J. R., Renz E. M., Cancio L. C., Panel P. O. C.-R. I. G. (2011). Prevention of infections associated with combat-related burn injuries. *The Journal of Trauma, Injury, Infection, and Critical Care*.

[71] Carr J. A., Phillips B. D., Bowling W. M. (2009). The utility of bronchoscopy after inhalation injury complicated by pneumonia in burn patients: results from the National Burn Repository. *Journal of Burn Care and Research: Official Publication of the American Burn Association*.

[72] Wahl W. L., Taddonio M. A., Arbabi S., Hemmila M. R. (2009). Duration of antibiotic therapy for ventilator-associated pneumonia in burn patients. *Journal of Burn Care and Research*.

[73] Sjöberg T., Mzezewa S., Jönsson K., Robertson V., Salemark L. (2003). Comparison of surface swab cultures and quantitative tissue biopsy cultures to predict sepsis in burn patients: a prospective study. *Journal of Burn Care & Rehabilitation*.

[74] Lavrentieva A., Kontakiotis T., Lazaridis L. (2007). Inflammatory markers in patients with severe burn injury: what is the best indicator of sepsis?. *Burns*.

[75] Ganatra M. A., Ganatra H. A. (2007). Method of quantitative bacterial count in burn wound. *Pakistan Journal of Medical Sciences*.

[76] Rondas A. A., Schols J. M., Halfens R. J., Stobberingh E. E. (2013). Swab versus biopsy for the diagnosis of chronic infected wounds. *Advances in Skin & Wound Care*.

[77] Angel D. E., Lloyd P., Carville K., Santamaria N. (2011). The clinical efficacy of two semi‐quantitative wound‐swabbing techniques in identifying the causative organism (s) in infected cutaneous wounds. *International Wound Journal*.

[78] Williams H. B., Breidenbach W. C., Callaghan W. B., Richards G. K., Prentis J. J. (1984). Are burn wound biopsies obsolete? A comparative study of bacterial quantitation in burn patients using the absorbent disc and biopsy techniques. *Annals of Plastic Surgery*.

[79] Cabral L., Afreixo V., Almeida L., Paiva J. A. (2016). The use of procalcitonin (PCT) for diagnosis of sepsis in burn patients: a meta-analysis. *PLoS One*.

[80] Lavrentieva A., Kontou P., Soulountsi V., Kioumis J., Chrysou O., Bitzani M. (2015). Implementation of a procalcitonin-guided algorithm for antibiotic therapy in the burn intensive care unit. *Annals of Burns and Fire Disasters*.

[81] Jr B. A. P., McManus A. T., Kim S. H., Goodwin C. W. (1998). Burn wound infections: current status. *World Journal of Surgery*.

[82] Mermel L. A., Allon M., Bouza E. (2009). Clinical practice guidelines for the diagnosis and management of intravascular catheter-related infection: 2009 Update by the Infectious Diseases Society of America. *Clinical Infectious Diseases*.

[83] Binkley S., Fishman N. O., LaRosa L. A. (2006). Comparison of unit-specific and hospital-wide antibiograms potential implications for selection of empirical antimicrobial therapy. *Infection Control & Hospital Epidemiology*.

[84] Barlam T. F., Cosgrove S. E., Abbo L. M. (2016). Implementing an antibiotic stewardship program: guidelines by the infectious diseases society of America and the society for healthcare Epidemiology of America. *Clinical Infectious Diseases*.

[85] Wagner B., Filice G. A., Drekonja D. (2014). Antimicrobial stewardship programs in inpatient hospital settings: a systematic review. *Infection Control & Hospital Epidemiology*.

[86] Abdulameer H., Swartzendruber J., Dennis A. (2018). 528 Combination topical therapy in burns and wounds may not be as symbiotic as once thought. *Journal of Burn Care and Research*.

[87] Cota J. M., FakhriRavari A., Rowan M. P., Chung K. K., Murray C. K., Akers K. S. (2016). Intravenous antibiotic and antifungal agent pharmacokinetic-pharmacodynamic dosing in adults with severe burn injury. *Clinical Therapeutics*.

[88] Mihai M. M., Dima M. B., Dima B., Holban A. M. (2019). Nanomaterials for wound healing and infection control. *Materials*.

[89] Gao W., Chen Y., Zhang Y., Zhang Q., Zhang L. (2018). Nanoparticle-based local antimicrobial drug delivery. *Advanced Drug Delivery Reviews*.

[90] Schneider M., Stracke F., Hansen S., Schaefer U. F. (2009). Nanoparticles and their interactions with the dermal barrier. *Dermato-Endocrinology*.

[91] Tiwari N., Kumar D., Priyadarshani A. (2023). Recent progress in polymeric biomaterials and their potential applications in skin regeneration and wound care management. *Journal of Drug Delivery Science and Technology*.

[92] Mordorski B., Prow T. (2016). Nanomaterials for wound healing. *Current Dermatology Reports*.

[93] Giordani B., Costantini P. E., Fedi S. (2019). Liposomes containing biosurfactants isolated from Lactobacillus gasseri exert antibiofilm activity against methicillin resistant *Staphylococcus aureus* strains. *European Journal of Pharmaceutics and Biopharmaceutics*.

[94] Omolo C. A., Megrab N. A., Kalhapure R. S. (2021). Liposomes with pH responsive ‘on and off’switches for targeted and intracellular delivery of antibiotics. *Journal of Liposome Research*.

[95] Rukavina Z., Šegvić Klarić M., Filipović-Grčić J., Lovrić J., Vanić Ž. (2018). Azithromycin-loaded liposomes for enhanced topical treatment of methicillin-resistant Staphyloccocus aureus (MRSA) infections. *International Journal of Pharmaceutics*.

[96] Jiang H., Xiong M., Bi Q., Wang Y., Li C. (2016). Self-enhanced targeted delivery of a cell wall–and membrane-active antibiotics, daptomycin, against staphylococcal pneumonia. *Acta Pharmaceutica Sinica B*.

[97] Lam A. H. C., Sandoval N., Wadhwa R. (2016). Assessment of free fatty acids and cholesteryl esters delivered in liposomes as novel class of antibiotic. *BMC Research Notes*.

[98] González-Paredes A., Sitia L., Ruyra A. (2019). Solid lipid nanoparticles for the delivery of anti-microbial oligonucleotides. *European Journal of Pharmaceutics and Biopharmaceutics*.

[99] Mhango E. K., Kalhapure R. S., Jadhav M. (2017). Preparation and optimization of meropenem-loaded solid lipid nanoparticles: in Vitro evaluation and molecular modeling. *AAPS PharmSciTech*.

[100] Farooq U., Ahmad T., Khan A. (2019). <p>Rifampicin conjugated silver nanoparticles: a new arena for development of antibiofilm potential against methicillin resistant<em> *Staphylococcus aureus*</em> and<em> *Klebsiella pneumoniae*</em></p&gt. *International Journal of Nanomedicine*.

[101] Figueiredo E., Ribeiro J., Nishio E. (2019). <p>New approach for simvastatin as an antibacterial: synergistic effect with bio-synthesized silver nanoparticles against multidrug-resistant bacteria</p&gt. *International Journal of Nanomedicine*.

[102] Halawani E. M., Hassan A. M., Gad El-Rab S. M. (2020). <p>Nanoformulation of biogenic cefotaxime-conjugated-silver nanoparticles for enhanced antibacterial efficacy against multidrug-resistant bacteria and anticancer studies</p&gt. *International Journal of Nanomedicine*.

[103] Esmaeillou M., Zarrini G., Ahangarzadeh Rezaee M., Shahbazi mojarrad J., Bahadori A. (2017). Vancomycin capped with silver nanoparticles as an antibacterial agent against multi-drug resistance bacteria. *Advanced Pharmaceutical Bulletin*.

[104] Iram S., Akbar Khan J., Aman N., Nadhman A., Zulfiqar Z., Arfat Yameen M. (2016). Enhancing the anti-enterococci activity of different antibiotics by combining with metal oxide nanoparticles. *Jundishapur Journal of Microbiology*.

[105] Zendegani E., Dolatabadi S. (2020). The efficacy of imipenem conjugated with synthesized silver nanoparticles against Acinetobacter baumannii clinical isolates, Iran. *Biological Trace Element Research*.

[106] Wan G., Ruan L., Yin Y., Yang T., Ge M., Cheng X. (2016). Effects of silver nanoparticles in combination with antibiotics on the resistant bacteria <em>Acinetobacter baumannii</em&gt. *International Journal of Nanomedicine*.

[107] Zhang Y., Zhang J., Chen W. (2017). Erythrocyte membrane-coated nanogel for combinatorial antivirulence and responsive antimicrobial delivery against *Staphylococcus aureus* infection. *Journal of Controlled Release*.

[108] Jiang C., Scholle F., Ghiladi R. A. (2019). Mn-doped Zn/S quantum dots as photosensitizers for antimicrobial photodynamic inactivation. *Photonic Diagnosis and Treatment of Infections and Inflammatory Diseases II*.

[109] Zhao C., Wu L., Wang X. (2020). Quaternary ammonium carbon quantum dots as an antimicrobial agent against gram-positive bacteria for the treatment of MRSA-infected pneumonia in mice. *Carbon*.

[110] Courtney C. M., Goodman S. M., McDaniel J. A., Madinger N. E., Chatterjee A., Nagpal P. (2016). Photoexcited quantum dots for killing multidrug-resistant bacteria. *Nature Materials*.

[111] Blanco-Fernandez B., Castaño O., Mateos-Timoneda M. Á., Engel E., Pérez-Amodio S. (2021). Nanotechnology approaches in chronic wound healing. *Advances in Wound Care*.

[112] Kalashnikova I., Das S., Seal S. (2015). Nanomaterials for wound healing: scope and advancement. *Nanomedicine*.

[113] Mendes C., Thirupathi A., Corrêa M. E., Gu Y., Silveira P. C. (2022). The use of metallic nanoparticles in wound healing: new perspectives. *International Journal of Molecular Sciences*.

[114] Roto R., Yusran Y., Kuncaka A. (2016). Magnetic adsorbent of Fe3O4@ SiO2 core-shell nanoparticles modified with thiol group for chloroauric ion adsorption. *Applied Surface Science*.

[115] Gul S., Khan S. B., Rehman I. U., Khan M. A., Khan M. (2019). A comprehensive review of magnetic nanomaterials modern day theranostics. *Frontiers in Materials*.

[116] Vijayakumar V., Samal S. K., Mohanty S., Nayak S. K. (2019). Recent advancements in biopolymer and metal nanoparticle-based materials in diabetic wound healing management. *International Journal of Biological Macromolecules*.

[117] Kwiatkowska A., Granicka L. H., Grzeczkowicz A. (2018). Gold nanoparticle-modified poly (vinyl chloride) surface with improved antimicrobial properties for medical devices. *Journal of Biomedical Nanotechnology*.

[118] Babushkina I., Gladkova E., Belova S., Norkin I. (2017). Application of preparations containing copper nanoparticles for the treatment of experimental septic wounds. *Bulletin of Experimental Biology and Medicine*.

[119] Kadiyala U., Turali-Emre E. S., Bahng J. H., Kotov N. A., Vanepps J. S. (2018). Unexpected insights into antibacterial activity of zinc oxide nanoparticles against methicillin resistant *Staphylococcus aureus* (MRSA). *Nanoscale*.

[120] Kedziora A., Speruda M., Krzyzewska E., Rybka J., Lukowiak A., Bugla-Ploskonska G. (2018). Similarities and differences between silver ions and silver in nanoforms as antibacterial agents. *International Journal of Molecular Sciences*.

[121] Li H., Gao Y., Li C., Ma G., Shang Y., Sun Y. (2016). A comparative study of the antibacterial mechanisms of silver ion and silver nanoparticles by Fourier transform infrared spectroscopy. *Vibrational Spectroscopy*.

[122] Kumar S. S. D., Rajendran N. K., Houreld N. N., Abrahamse H. (2018). Recent advances on silver nanoparticle and biopolymer-based biomaterials for wound healing applications. *International Journal of Biological Macromolecules*.

[123] Deepachitra R., Lakshmi R. P., Sivaranjani K., Chandra J. H., Sastry T. P. (2015). Nanoparticles embedded biomaterials in wound treatment: a review. *Journal of Chemical and Pharmaceutical Sciences*.

[124] Lansdown A. B., Mirastschijski U., Stubbs N., Scanlon E., Ågren M. S. (2007). Zinc in wound healing: theoretical, experimental, and clinical aspects. *Wound Repair and Regeneration*.

[125] Pivodová V., Franková J., Galandáková A., Ulrichová J. (2015). In vitro AuNPs’ cytotoxicity and their effect on wound healing. *Nanobiomedicine*.

[126] Guo Z., Chen Y., Wang Y., Jiang H., Wang X. (2020). Advances and challenges in metallic nanomaterial synthesis and antibacterial applications. *Journal of Materials Chemistry B*.

[127] Chandrakala V., Aruna V., Angajala G. (2022). Review on metal nanoparticles as nanocarriers: current challenges and perspectives in drug delivery systems. *Emergent Materials*.

[128] Lipinski C. A. (2004). Lead-and drug-like compounds: the rule-of-five revolution. *Drug Discovery Today: Technologies*.

[129] Eleraky N. E., Allam A., Hassan S. B., Omar M. M. (2020). Nanomedicine fight against antibacterial resistance: an overview of the recent pharmaceutical innovations. *Pharmaceutics*.

[130] Ferreira M., Aguiar S., Bettencourt A., Gaspar M. M. (2021). Lipid-based nanosystems for targeting bone implant-associated infections: current approaches and future endeavors. *Drug Delivery and Translational Research*.

[131] Abed N., Couvreur P. (2014). Nanocarriers for antibiotics: a promising solution to treat intracellular bacterial infections. *International Journal of Antimicrobial Agents*.

[132] Hajiahmadi F., Alikhani M. Y., Shariatifar H., Arabestani M. R., Ahmadvand D. (2019). <p>The bactericidal effect of lysostaphin coupled with liposomal vancomycin as a dual combating system applied directly on methicillin-resistant <em>*Staphylococcus aureus*</em> infected skin wounds in mice </p&gt. *International Journal of Nanomedicine*.

[133] Ferreira M., Ogren M., Dias J. N. (2021). Liposomes as antibiotic delivery systems: a promising nanotechnological strategy against antimicrobial resistance. *Molecules*.

[134] Alemdaroğlu C., Degim Z., Celebi N., Şengezer M., Alömeroglu M., Nacar A. (2008). Investigation of epidermal growth factor containing liposome formulation effects on burn wound healing. *Journal of Biomedical Materials Research Part A*.

[135] Price C., Horton J., Baxter C. (1992). Liposome delivery of aminoglycosides in burn wounds. *Surgery Gynecology & Obstetrics*.

[136] Cheng R., Liu L., Xiang Y. (2020). Advanced liposome-loaded scaffolds for therapeutic and tissue engineering applications. *Biomaterials*.

[137] Li D., van Nostrum C. F., Mastrobattista E., Vermonden T., Hennink W. E. (2017). Nanogels for intracellular delivery of biotherapeutics. *Journal of Controlled Release*.

[138] Keskin D., Zu G., Forson A. M., Tromp L., Sjollema J., van Rijn P. (2021). Nanogels: a novel approach in antimicrobial delivery systems and antimicrobial coatings. *Bioactive Materials*.

[139] Mura S., Nicolas J., Couvreur P. (2013). Stimuli-responsive nanocarriers for drug delivery. *Nature Materials*.

[140] Molina M., Asadian-Birjand M., Balach J., Bergueiro J., Miceli E., Calderón M. (2015). Stimuli-responsive nanogel composites and their application in nanomedicine. *Chemical Society Reviews*.

[141] Plamper F. A., Richtering W. (2017). Functional microgels and microgel systems. *Accounts of Chemical Research*.

[142] Anjum S., Gupta B. (2018). Bioengineering of functional nanosilver nanogels for smart healthcare systems. *Global Challenges*.

[143] Chen T., Li Q., Guo L. (2016). Lower cytotoxicity, high stability, and long-term antibacterial activity of a poly (methacrylic acid)/isoniazid/rifampin nanogel against multidrug-resistant intestinal *Mycobacterium tuberculosis*. *Materials Science and Engineering: C*.

[144] Wu T., Liao W., Wang W. (2018). Genipin-crosslinked carboxymethyl chitosan nanogel for lung-targeted delivery of isoniazid and rifampin. *Carbohydrate Polymers*.

[145] Chen T., Chen L., Li H. (2014). Design and in vitro evaluation of a novel poly (methacrylic acid)/metronidazole antibacterial nanogel as an oral dosage form. *Colloids and Surfaces B: Biointerfaces*.

[146] Chen Y.-F., Chen G.-Y., Chang C.-H. (2019). TRAIL encapsulated to polypeptide-crosslinked nanogel exhibits increased anti-inflammatory activities in Klebsiella pneumoniae-induced sepsis treatment. *Materials Science and Engineering: C*.

[147] Kłodzińska S. N., Molchanova N., Franzyk H., Hansen P. R., Damborg P., Nielsen H. M. (2018). Biopolymer nanogels improve antibacterial activity and safety profile of a novel lysine-based* α*-peptide/*β*-peptoid peptidomimetic. *European Journal of Pharmaceutics and Biopharmaceutics*.

[148] Nordström R., Browning K. L., Parra-Ortiz E. (2020). Membrane interactions of antimicrobial peptide-loaded microgels. *Journal of Colloid and Interface Science*.

[149] Parilti R., Caprasse J., Riva R. (2018). Antimicrobial peptide encapsulation and sustained release from polymer network particles prepared in supercritical carbon dioxide. *Journal of Colloid and Interface Science*.

[150] Li L.-L., Xu J.-H., Qi G.-B., Zhao X., Yu F., Wang H. (2014). Core–shell supramolecular gelatin nanoparticles for adaptive and “on-demand” antibiotic delivery. *ACS Nano*.

[151] Xiong M. H., Li Y. J., Bao Y., Yang X. Z., Hu B., Wang J. (2012). Bacteria‐responsive multifunctional nanogel for targeted antibiotic delivery. *Advanced Materials*.

[152] Xiong M.-H., Bao Y., Yang X.-Z., Wang Y.-C., Sun B., Wang J. (2012). Lipase-sensitive polymeric triple-layered nanogel for “on-demand” drug delivery. *Journal of the American Chemical Society*.

[153] Sproul E. P., Nandi S., Chee E. (2020). Development of biomimetic antimicrobial platelet-like particles comprised of microgel nanogold composites. *Regenerative engineering and translational medicine*.

[154] El-Feky G. S., El-Banna S. T., El-Bahy G., Abdelrazek E., Kamal M. (2017). Alginate coated chitosan nanogel for the controlled topical delivery of Silver sulfadiazine. *Carbohydrate Polymers*.

[155] Martínez-Higuera A., Rodríguez-Beas C., Villalobos-Noriega J. M. A. (2021). Hydrogel with silver nanoparticles synthesized by Mimosa tenuiflora for second-degree burns treatment. *Scientific Reports*.

[156] D’Souza A., Yoon J. H., Beaman H. (2020). Nine-residue peptide self-assembles in the presence of silver to produce a self-healing, cytocompatible, antimicrobial hydrogel. *ACS Applied Materials & Interfaces*.

[157] Chakrabarti S., Mazumder B., Rajkonwar J., Pathak M. P., Patowary P., Chattopadhyay P. (2021). bFGF and collagen matrix hydrogel attenuates burn wound inflammation through activation of ERK and TRK pathway. *Scientific Reports*.

[158] Li M., Jiang X., Wang D., Xu Z., Yang M. (2019). In situ reduction of silver nanoparticles in the lignin based hydrogel for enhanced antibacterial application. *Colloids and Surfaces B: Biointerfaces*.

[159] Alarcon E. I., Udekwu K. I., Noel C. W. (2015). Safety and efficacy of composite collagen–silver nanoparticle hydrogels as tissue engineering scaffolds. *Nanoscale*.

[160] Oliveira R., Rouzé R., Quilty B. (2014). Mechanical properties and in vitro characterization of polyvinyl alcohol-nano-silver hydrogel wound dressings. *Interface focus*.

[161] Masood N., Ahmed R., Tariq M. (2019). Silver nanoparticle impregnated chitosan-PEG hydrogel enhances wound healing in diabetes induced rabbits. *International Journal of Pharmaceutics*.

[162] Rajendiran K., Zhao Z., Pei D.-S., Fu A. (2019). Antimicrobial activity and mechanism of functionalized quantum dots. *Polymers*.

[163] Lim S. Y., Shen W., Gao Z. (2015). Carbon quantum dots and their applications. *Chemical Society Reviews*.

[164] Knoblauch R., Geddes C. D. (2020). Carbon nanodots in photodynamic antimicrobial therapy: a review. *Materials*.

[165] Yougbaré S., Mutalik C., Krisnawati D. I. (2020). Nanomaterials for the photothermal killing of bacteria. *Nanomaterials*.

[166] Tim M. (2015). Strategies to optimize photosensitizers for photodynamic inactivation of bacteria. *Journal of Photochemistry and Photobiology B: Biology*.

[167] Chen Y., Gao Y., Chen Y., Liu L., Mo A., Peng Q. (2020). Nanomaterials-based photothermal therapy and its potentials in antibacterial treatment. *Journal of Controlled Release*.

[168] Gao S., Yan X., Xie G. (2019). Membrane intercalation-enhanced photodynamic inactivation of bacteria by a metallacycle and TAT-decorated virus coat protein. *Proceedings of the National Academy of Sciences*.

[169] Arana L., Gallego L., Alkorta I. (2021). Incorporation of antibiotics into solid lipid nanoparticles: a promising approach to reduce antibiotic resistance emergence. *Nanomaterials*.

[170] Hosseini S. M., Farmany A., Abbasalipourkabir R., Soleimani Asl S., Nourian A., Arabestani M. R. (2019). Doxycycline-encapsulated solid lipid nanoparticles for the enhanced antibacterial potential to treat the chronic brucellosis and preventing its relapse: in vivo study. *Annals of Clinical Microbiology and Antimicrobials*.

[171] Ghaderkhani J., Yousefimashouf R., Arabestani M., Roshanaei G., Asl S. S., Abbasalipourkabir R. (2019). Improved antibacterial function of Rifampicin-loaded solid lipid nanoparticles on Brucella abortus. *Artificial Cells, Nanomedicine, and Biotechnology*.

[172] Prastiwi N., Lesbatta K., Lesbatta K. (2021). Application of solid lipid nanoparticles preparation in infection caused by antibiotic-resistant bacteria. *Indonesian Journal of Pharmacology and Therapy*.

[173] Ling Z., Yonghong L., Junfeng L., Li Z., Xianqiang L. (2018). Tilmicosin‐and florfenicol‐loaded hydrogenated castor oil‐solid lipid nanoparticles to pigs: combined antibacterial activities and pharmacokinetics. *Journal of Veterinary Pharmacology and Therapeutics*.

[174] Ghaffari S., Alihosseini F., Rezayat Sorkhabadi S. M. (2018). Nanotechnology in wound healing; semisolid dosage forms containing curcumin-ampicillin solid lipid nanoparticles, in-vitro, ex-vivo and in-vivo characteristics. *Advanced Pharmaceutical Bulletin*.

[175] Farmoudeh A., enayatifard R., Saeedi M. (2022). Methylene blue loaded solid lipid nanoparticles: preparation, optimization, and in-vivo burn healing assessment. *Journal of Drug Delivery Science and Technology*.

[176] Sandri G., Bonferoni M. C., D’Autilia F. (2013). Wound dressings based on silver sulfadiazine solid lipid nanoparticles for tissue repairing. *European Journal of Pharmaceutics and Biopharmaceutics*.

